# Supplementary material for: Objective hearing threshold identification from auditory brainstem response measurements using supervised and self-supervised approaches
Source: BMC Neurosci. 2022 Dec 27;23:81. doi: 10.1186/s12868-022-00758-0 (PMC9795643; doi:10.1186/s12868-022-00758-0)
Supplement: Supplementary file 1 — Additional file 1: S1. Neural network model architectures. S2. Evaluation curves. S3. Volcano plots of GMC mutant lines. S4. Comparison of top click candidate genes with hearing threshold changes. S5. Comparison of top 30 kHz candidate genes with hearing threshold changes [file 12868_2022_758_MOESM1_ESM.pdf]

Supplement 1 - Neural network model architectures

Model I

Using a 1000 time step input vector of any stimulus frequency, this model predicts:

- response yes/no (0/1)
- the frequency of the stimulus (click, 6, 12, 18, 24, 30 kHz)
- the sound level of the stimulus (5, 10, ..., 95 dB)

```
__________________________________________________________________________________________________
Layer (type)                    Output Shape         Param #     Connected to
==================================================================================================
input_3 (InputLayer)            (None, 1000, 1)      0
__________________________________________________________________________________________________
batch_normalization_v1_13 (Batc (None, 1000, 1)      4           input_3[0][0]
__________________________________________________________________________________________________
conv1d_11 (Conv1D)              (None, 1000, 256)    65792       batch_normalization_v1_13[0][0]
__________________________________________________________________________________________________
batch_normalization_v1_14 (Batc (None, 1000, 256)    1024        conv1d_11[0][0]
__________________________________________________________________________________________________
activation_11 (Activation)      (None, 1000, 256)    0           batch_normalization_v1_14[0][0]
__________________________________________________________________________________________________
conv1d_12 (Conv1D)              (None, 1000, 128)    4194432     activation_11[0][0]
__________________________________________________________________________________________________
batch_normalization_v1_15 (Batc (None, 1000, 128)    512         conv1d_12[0][0]
__________________________________________________________________________________________________
activation_12 (Activation)      (None, 1000, 128)    0           batch_normalization_v1_15[0][0]
__________________________________________________________________________________________________
average_pooling1d (AveragePooli (None, 250, 128)     0           activation_12[0][0]
__________________________________________________________________________________________________
dropout_5 (Dropout)             (None, 250, 128)     0           average_pooling1d[0][0]
__________________________________________________________________________________________________
conv1d_13 (Conv1D)              (None, 250, 64)      524352      dropout_5[0][0]
__________________________________________________________________________________________________
batch_normalization_v1_16 (Batc (None, 250, 64)      256         conv1d_13[0][0]
__________________________________________________________________________________________________
activation_13 (Activation)      (None, 250, 64)      0           batch_normalization_v1_16[0][0]
__________________________________________________________________________________________________
conv1d_14 (Conv1D)              (None, 250, 32)      65568       activation_13[0][0]
__________________________________________________________________________________________________
batch_normalization_v1_17 (Batc (None, 250, 32)      128         conv1d_14[0][0]
__________________________________________________________________________________________________
activation_14 (Activation)      (None, 250, 32)      0           batch_normalization_v1_17[0][0]
__________________________________________________________________________________________________
average_pooling1d_1 (AveragePoo (None, 125, 32)      0           activation_14[0][0]
__________________________________________________________________________________________________
dropout_6 (Dropout)             (None, 125, 32)      0           average_pooling1d_1[0][0]
__________________________________________________________________________________________________
conv1d_15 (Conv1D)              (None, 125, 16)      8208        dropout_6[0][0]
__________________________________________________________________________________________________
batch_normalization_v1_18 (Batc (None, 125, 16)      64          conv1d_15[0][0]
__________________________________________________________________________________________________
activation_15 (Activation)      (None, 125, 16)      0           batch_normalization_v1_18[0][0]
__________________________________________________________________________________________________
conv1d_16 (Conv1D)              (None, 125, 8)       1032        activation_15[0][0]
__________________________________________________________________________________________________
batch_normalization_v1_19 (Batc (None, 125, 8)       32          conv1d_16[0][0]
__________________________________________________________________________________________________
activation_16 (Activation)      (None, 125, 8)       0           batch_normalization_v1_19[0][0]
__________________________________________________________________________________________________
average_pooling1d_2 (AveragePoo (None, 62, 8)        0           activation_16[0][0]
__________________________________________________________________________________________________
dropout_7 (Dropout)             (None, 62, 8)        0           average_pooling1d_2[0][0]
__________________________________________________________________________________________________
conv1d_17 (Conv1D)              (None, 62, 4)        132         dropout_7[0][0]
__________________________________________________________________________________________________
batch_normalization_v1_20 (Batc (None, 62, 4)        16          conv1d_17[0][0]
__________________________________________________________________________________________________
activation_17 (Activation)      (None, 62, 4)        0           batch_normalization_v1_20[0][0]
__________________________________________________________________________________________________
conv1d_18 (Conv1D)              (None, 62, 1)        9           activation_17[0][0]
__________________________________________________________________________________________________
batch_normalization_v1_21 (Batc (None, 62, 1)        4           conv1d_18[0][0]
__________________________________________________________________________________________________
activation_18 (Activation)      (None, 62, 1)        0           batch_normalization_v1_21[0][0]
__________________________________________________________________________________________________
flatten_2 (Flatten)             (None, 62)           0           activation_18[0][0]
```

```
__________________________________________________________________________________________________
dense_2 (Dense)                 (None, 32)           2016        flatten_2[0][0]
__________________________________________________________________________________________________
main_prediction (Dense)         (None, 1)            33          dense_2[0][0]
__________________________________________________________________________________________________
frequency_prediction (Dense)    (None, 6)            198         dense_2[0][0]
__________________________________________________________________________________________________
sl_prediction (Dense)           (None, 20)           660         dense_2[0][0]
==================================================================================================
Total params: 4,864,472
Trainable params: 4,863,452
Non-trainable params: 1,020
```

Model II

Using an input vector of 20 sound level prediction scores from model I output, this model predicts:

- the frequency of the stimulus (click, 6, 12, 18, 24, 30 kHz)
- the hearing threshold (5, 10, ..., 95 dB)

```
__________________________________________________________________________________________________
Layer (type)                    Output Shape         Param #     Connected to
==================================================================================================
input_2 (InputLayer)            (None, 20, 1)        0
__________________________________________________________________________________________________
batch_normalization_v1_4 (Batch (None, 20, 1)        4           input_2[0][0]
__________________________________________________________________________________________________
conv1d_3 (Conv1D)               (None, 20, 128)      896         batch_normalization_v1_4[0][0]
__________________________________________________________________________________________________
batch_normalization_v1_5 (Batch (None, 20, 128)      512         conv1d_3[0][0]
__________________________________________________________________________________________________
activation_3 (Activation)       (None, 20, 128)      0           batch_normalization_v1_5[0][0]
__________________________________________________________________________________________________
conv1d_4 (Conv1D)               (None, 20, 64)       41024       activation_3[0][0]
__________________________________________________________________________________________________
batch_normalization_v1_6 (Batch (None, 20, 64)       256         conv1d_4[0][0]
__________________________________________________________________________________________________
activation_4 (Activation)       (None, 20, 64)       0           batch_normalization_v1_6[0][0]
__________________________________________________________________________________________________
max_pooling1d_1 (MaxPooling1D)  (None, 6, 64)        0           activation_4[0][0]
__________________________________________________________________________________________________
dropout_2 (Dropout)             (None, 6, 64)        0           max_pooling1d_1[0][0]
__________________________________________________________________________________________________
conv1d_5 (Conv1D)               (None, 6, 32)        8224        dropout_2[0][0]
__________________________________________________________________________________________________
batch_normalization_v1_7 (Batch (None, 6, 32)        128         conv1d_5[0][0]
__________________________________________________________________________________________________
activation_5 (Activation)       (None, 6, 32)        0           batch_normalization_v1_7[0][0]
__________________________________________________________________________________________________
conv1d_6 (Conv1D)               (None, 6, 16)        1552        activation_5[0][0]
__________________________________________________________________________________________________
batch_normalization_v1_8 (Batch (None, 6, 16)        64          conv1d_6[0][0]
__________________________________________________________________________________________________
activation_6 (Activation)       (None, 6, 16)        0           batch_normalization_v1_8[0][0]
__________________________________________________________________________________________________
flatten_1 (Flatten)             (None, 96)           0           activation_6[0][0]
__________________________________________________________________________________________________
dense_1 (Dense)                 (None, 64)           6208        flatten_1[0][0]
__________________________________________________________________________________________________
main_prediction (Dense)         (None, 21)           1365        dense_1[0][0]
__________________________________________________________________________________________________
frequency_prediction (Dense)    (None, 6)            390         dense_1[0][0]
==================================================================================================
Total params: 60,623
Trainable params: 60,141
Non-trainable params: 482
```

Supplement 2 - Evaluation curves

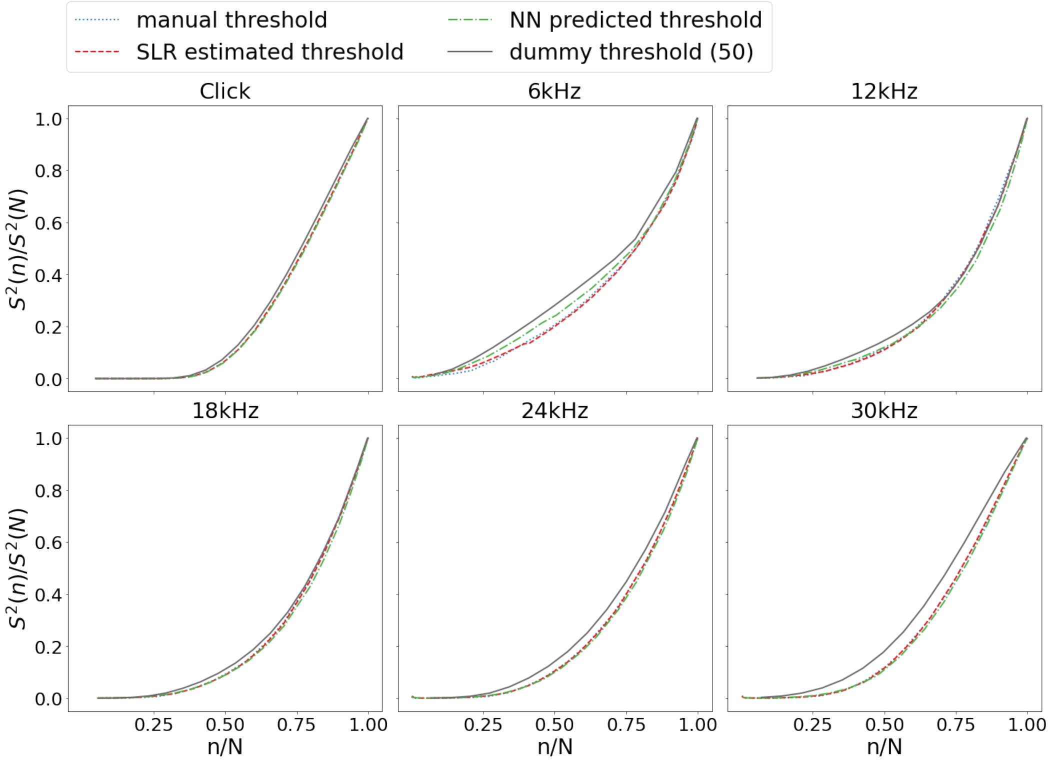

(a) NN/SLR GMC-ING (experiments 2 and 6)

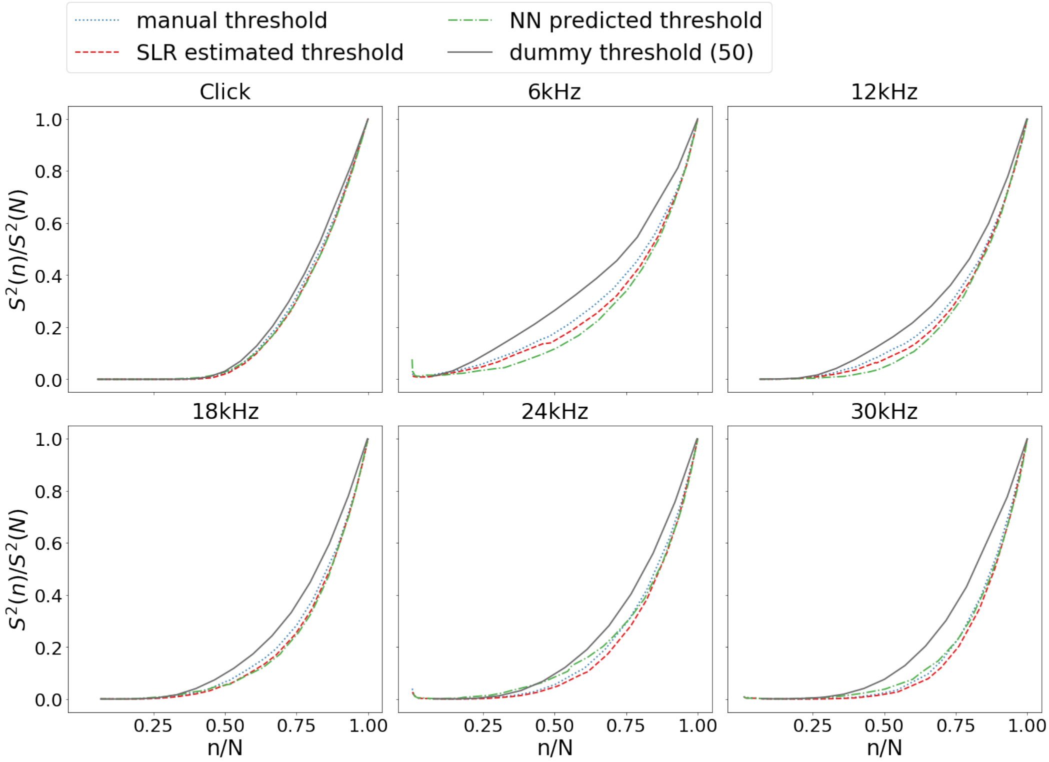

(b) NN/SLR ING-GMC (experiments 3 and 7)

Fig. S1: Objective comparison of the quality of threshold finding methods using evaluation curves. Evaluation curves allow the relative comparison of threshold finding methods without requiring absolute ground truth labels. Here, four methods are compared: manual thresholds (blue, dotted lines), SLR estimations (red, dashed lines), NN predictions (green, dash-dotted lines), and a "always 50 dB" dummy method (grey, solid lines).

Fig. S1: (continued from previous page)

Separate plots show evaluation curves for each stimulus (click, 6 kHz - 30 kHz). Plots show the normalized time variance of the averaged signal $S^2(n)/S^2(N)$ (y-axis) vs. the total percentage of ABR curves included in the cumulative average n/N (x-axis). a) shows NN predictions and SLR estimations from experiments 2 and 6, b) shows NN predictions and SLR estimations from experiments 3 and 7, as introduced in table 2. Two methods can be compared in a way that the evaluation curve of the better method deviates from zero later. Ideally, the curve of the best method is always below all other curves. Strangely, when GMC-trained/calibrated models where tested on ING data (experiments 2 and 6), human, NN and SLR were not much better than the dummy method. In addition, except for the 6 kHz stimulus, they did not differ much from each other. In contrast, when ING-trained/calibrated models were tested on GMC data (experiments 3 and 7), NN and SLR models were mostly better than the human method, with NN being best for click and 6 kHz to 18 kHz and SLR being best for 24 kHz and 30 kHz.

Supplement 3 - Volcano plots of GMC mutant lines

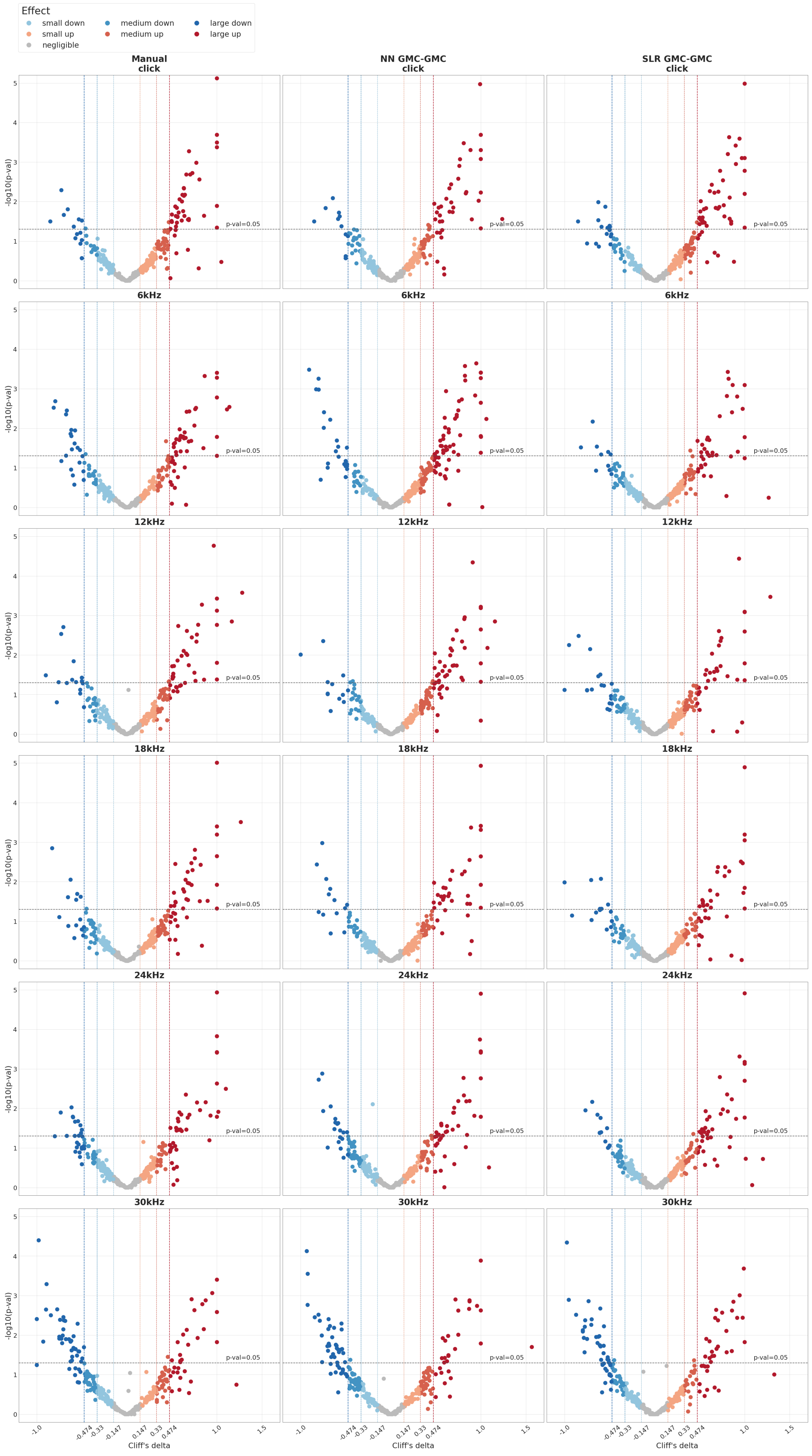

Fig. S2: (continued from previous page)
Biologically relevant changes in hearing thresholds - GMC lines, all stimuli. For each GMC line, Volcano plots show significance vs. relevance for comparisons of mutant and control mice. For each mouse line, represented by a dot, hearing thresholds were used to calculate significance (Wilcoxon test, y-axis) and non-parametric effect size (Cliff's delta [39], x-axis) of mutant vs. control animals. Vertical lines indicate margins for small (0.147), medium (0.33) and large (0.474) effects as suggested in [38]. The horizontal line indicates the 0.05 significance threshold level. Accordingly, mutant lines represented by data points in the upper left and upper right areas denote lines with significant as well as relevant changes and thus are considered worthwhile candidates (see supplement table S1). Dot colors in addition represent effect size as shown in the legend. Plot rows represent different stimuli (click, 6 kHz - 30 kHz). Columns compare the three hearing threshold finding methods compared in this work (left: manual, middle: NN, right: SLR).

Supplement 4 - Comparison of top click candidate genes with hearing threshold changes

Table S1: Comparison of top candidate genes with modified click hearing threshold. The columns show genes with a strong effect size for mutant-control group comparisons of click hearing thresholds. In each column, genes are ordered by non-parametric effect size (Cliff's delta [39]), in descending order. Only genes with a significant ($p < 0.05$) and large effect size ($|d| > 0.474$, [38]) in any of the three methods are shown. Column headers indicate the direction of the effect and the method that was used to identify the hearing thresholds, respectively. The "combined" column contains the combined list of all three methods.

**increased click threshold**				**decreased click threshold**			
**combined**	**manual**	**NN**	**SLR**	**combined**	**manual**	**NN**	**SLR**
Palm3	*Zfp280d*	*Slc20a2*	*Lsm1*	*Dio1*	*Dio1*	*Dio1*	*Dio1*
Zfp280d	*Lsm1*	*Prkd2*	*Strbp*	*Gstm6*	*Gstm6*	*Ucp1*	*Hnf4a*
Strbp	*Strbp*	*Zfp280d*	*Palm3*	*Hepacam2*	*Hepacam2*	*Cilp2*	*Gstt1*
Hipk3	*Hipk3*	*Strbp*	*Zfp280d*	*Slc25a15*	*Slc25a15*	*Gstm6*	*Ostf1*
Prkd2	*Palm3*	*Lsm1*	*Hipk3*	*Ostf1*	*Ostf1*	*Raet1c*	*Angptl3*
Lsm1	*Prkd2*	*Palm3*	*Prkd2*	*Ucp1*	*Rab35*	*Rab35*	*Phactr4*
Mipol1	*Mipol1*	*Hipk3*	*Plag1*	*Rab35*	*Cilp2*	*Slc25a15*	*Rab35*
Hoxa2	*Hoxa2*	*Mipol1*	*Hoxa2*	*Hnf4a*			*Cilp2*
Slc20a2	*Plag1*	*Vps13c*	*Aspa*	*Cilp2*			*Prox2*
Plag1	*Ldlr*	*Plag1*	*Dpp3*	*Prox2*			
Ldlr	*Hunk*	*Nacc1*	*Vps13c*	*Phactr4*			
Hunk	*Atp5g2*	*Btbd9*	*Mipol1*	*Raet1c*			
Atp5g2	*Ube3c*	*Alkbh6*	*Btbd9*	*Gstt1*			
Btbd9	*Btbd9*	*Zdhhc5*	*Alkbh6*	*Angptl3*			
Ube3c	*Nacc1*	*Rabgap1*	*Zdhhc5*				
Nacc1	*Tle1*	*Aspa*	*Bms1*				
Tle1	*Gpatch2l*	*Hoxa2*	*Nacc1*				
Gpatch2l	*Cidec*	*Sytl4*	*Plekha1*				
Cidec	*Alkbh6*	*Pdcd5*	*Ldlr*				
Alkbh6	*Spryd3*	*Ldlr*	*Adprm*				
Spryd3	*Bccip*	*Ttll12*	*Wrnip1*				
Bccip	*Pdcd5*	*Hunk*	*Sytl4*				
Pdcd5	*Ppp4r3b*	*Ppp4r3b*	*Hdac1*				
Ppp4r3b	*Csnk1g2*	*Kansl1l*	*Ppp4r3b*				
Csnk1g2	*Ngdn*	*Bccip*	*Tle1*				
Ngdn	*Gpsm2*	*Csnk1g2*	*Gpsm2*				
Gpsm2	*Aspa*	*Plekha1*	*Pkn2*				
Aspa	*Plekha1*	*Tle1*	*Me2*				
Pfkfb3	*Pfkfb3*	*Fdx1*	*Ttll12*				
Plekha1	*Wrnip1*	*Gpsm2*	*Rabgap1*				
Wrnip1	*Zdhhc5*	*Atp5g2*	*Csnk1g2*				
Zdhhc5	*Uggt2*		*Tbl1xr1*				
Dpp3	*Gdi2*		*Pdcd5*				
Uggt2	*Sec14l4*		*Hunk*				
Gdi2	*Tanc2*		*Atp5g2*				
Sec14l4	*Rfxank*		*Fdx1*				
Sytl4	*Me2*		*Tanc2*				
Rfxank	*Gsk3a*		*Cenpv*				
Tanc2			*Bccip*				
Me2							
Gsk3a							
Rabgap1							
Ttll12							
Fdx1							
Bms1							
Hdac1							
Tbl1xr1							
Adprm							
Vps13c							
Kansl1l							
Pkn2							
Cenpv							

Supplement 5 - Comparison of top 30 kHz candidate genes with hearing threshold changes

Table S2: Comparison of top candidate genes with modified 30 kHz hearing threshold. The columns show genes with a strong effect size for mutant-control group comparisons of 30 kHz hearing thresholds. In each column, genes are ordered by non-parametric effect size (Cliff's delta [39]), in descending order. Only genes with a significant ($p < 0.05$) and large effect size ($|d| > 0.474$, [38]) in any of the three methods are shown. Column headers indicate the direction of the effect and the method that was used to identify the hearing thresholds, respectively. The "combined" column contains the ranked combined list of all three methods.

**increased 30 kHz threshold**				**decreased 30 kHz threshold**			
**combined**	**manual**	**NN**	**SLR**	**combined**	**manual**	**NN**	**SLR**
Chst5	*Zfp280d*	*Chst5*	*Zfp280d*	*Miga1*	*Miga1*	*Gfpt2*	*Gfpt2*
Palm3	*Strbp*	*Zfp280d*	*Plag1*	*Gfpt2*	*Gfpt2*	*Rest*	*Hnf4a*
Strbp	*Palm3*	*Strbp*	*Strbp*	*Aqp6*	*Aqp6*	*Tap1*	*Rest*
Zfp280d	*Uggt2*	*Plag1*	*Ttll12*	*Rest*	*Rest*	*Hnf4a*	*Fgfr1op*
Uggt2	*Prkd2*	*Ube3c*	*Ube3c*	*Cotl1*	*Cotl1*	*Becn1*	*Msh5*
Prkd2	*Ube3c*	*Prkd2*	*Prkd2*	*Hnf4a*	*Hnf4a*	*Fbp2*	*Dis3*
Ube3c	*Plag1*	*Palm3*	*Palm3*	*Cenph*	*Cenph*	*Msh5*	*Miga1*
Plag1	*Nacc1*	*Uggt2*	*Mipol1*	*Dnajc27*	*Msh5*	*Aqp6*	*Fbp2*
Nacc1	*Gsk3a*	*Ttll12*	*Mthfsl*	*Msh5*	*Dnajc27*	*Etfdh*	*Becn1*
Gsk3a	*Hipk3*	*Gsk3a*	*Cnot6l*	*Lama1*	*Lama1*	*Ostf1*	*Dnajc27*
Hipk3	*Ttll12*	*Hipk3*	*Gsk3a*	*Pkig*	*Pkig*	*Cilp2*	*Ppy*
Ttll12	*Zdhhc5*	*Nacc1*	*Csnk1g2*	*Fgfr1op*	*Fgfr1op*	*Lss*	*Tap1*
Zdhhc5	*Ldlr*	*Rnf186*	*Rnf186*	*Fbp2*	*Fbp2*	*Gstm6*	*Fam162a*
Mipol1	*Ngdn*	*Adprm*	*Adprm*	*Cyp7a1*	*Cyp7a1*	*Pkig*	*Cenph*
Ldlr	*Adprm*	*Zdhhc5*	*Hipk3*	*Fam162a*	*Fam162a*	*Galk2*	*Cyp7a1*
Ngdn	*Pdcd5*	*Aldh1l1*	*Alkbh6*	*Galk2*	*Galk2*	*Mgat1*	*Galk2*
Adprm	*Rnf186*	*Csnk1g2*	*Aldh1l1*	*Yae1d1*	*Yae1d1*	*Slc25a15*	*Lss*
Rnf186	*Fabp2*	*Paox*	*Pdcd5*	*Becn1*	*Becn1*	*Ppy*	*Cotl1*
Pdcd5	*Entpd1*	*Cnot6l*	*Uggt2*	*Ly6g6d*	*Ly6g6d*	*Ctc1*	*Pdhx*
Fabp2	*Csnk1g2*	*Alkbh6*	*Nacc1*	*Jmjd6*	*Jmjd6*	*Sarnp*	*Vwa8*
Aldh1l1		*Fabp2*	*Paox*	*Vwa8*	*Dis3*	*Vwa8*	*Cilp2*
Entpd1			*Zdhhc5*	*Dis3*	*Vwa8*	*Cyp7a1*	*Zbtb24*
Cnot6l			*Gpsm2*	*Tap1*	*Tap1*	*Dnajc27*	*Mgat1*
Csnk1g2			*Npepps*	*Phactr4*	*Phactr4*	*Fgfr1op*	*Yae1d1*
Paox				*C1galt1*	*C1galt1*	*Nubp2*	*Lactb*
Mthfsl				*Cth*	*Cth*	*Cotl1*	*Sarnp*
Gpsm2				*Cilp2*	*Cilp2*	*Fam162a*	*Scmh1*
Npepps				*Ppy*	*Ppy*	*Pdhx*	*Rcn1*
Alkbh6				*Cdc123*	*Cdc123*	*Lactb*	*Nubp2*
				Scmh1	*Scmh1*	*Capn12*	
				Ndufa10	*Ndufa10*	*Yae1d1*	
				Pdhx	*Pdhx*	*Jmjd6*	
				Lss	*Lss*		
				Capn12	*Capn12*		
				Sarnp	*Sarnp*		
				Cystm1	*Cystm1*		
				Ube3a	*Ube3a*		
				Nubp2			
				Slc25a15			
				Zbtb24			
				Rcn1			
				Etfdh			
				Ostf1			
				Lactb			
				Ctc1			
				Mgat1			
				Gstm6			
